# Identification of potential crucial genes and biomarkers from neutrophils in sepsis using bioinformatics analysis

**DOI:** 10.1097/MD.0000000000041216

**Published:** 2025-01-03

**Authors:** Junfeng Zhang, Qinghui Fu, Jianfeng Zhao

**Affiliations:** a Department of Intensive Care Unit, Beilun People’s Hospital, Ningbo, Zhejiang Province, China; b Department of SICU, The First Affiliated Hospital, Zhejiang University School of Medicine, Hangzhou, Zhejiang Province, China.

**Keywords:** bioinformatics, biomarkers, diagnosis, sepsis

## Abstract

Sepsis is a complex series of immune responses to infection and is commonly associated with acquired immunodeficiency. The current study aimed to identify the biomarkers of sepsis. Differential expression analysis and protein–protein interaction analysis were conducted to explore potential biomarkers. Gene Ontology enrichment analysis and Kyoto Encyclopedia of Genes and Genomes analysis were performed to explore potential mechanisms. The Immune Cell Abundance Identifier website was utilized to evaluate immune cell infiltration in the whole blood. Receiver operating characteristic curve and area under the curve were applied to compare the prognostic accuracy of hub genes. Six genes were selected via differentially expressed gene analysis and protein–protein interaction analysis. Furthermore, CTSD, GADD45A, MAPK14, MMP9, and VIM were selected via validation analysis of independent datasets. Immune infiltration analysis showed that CTSD, GADD45A, MAPK14, MMP9, and VIM may regulate immune cells via neutrophils. Patients with sepsis had a significantly higher expression of CTSD, GADD45A, MAPK14, MMP9, and VIM than normal health controls. The area under the curve of CTSD, GADD45A, MAPK14, MMP9, and VIM were 0.90 (0.83–0.97), 0.89 (0.81–0.96), 0.91 (0.84–0.87), 0.95 (0.91–1.00), and 0.95 (0.91–1.00), respectively. According to the validation result of RT-PCR, only MAPK14 was significantly upregulated compared with controls, which was concordant with the bioinformatics analysis results. This study identified several potential diagnostic genes including CTSD, GADD45A, MAPK14, MMP9, and VIM. These genes may regulate the expression of immune cells via neutrophils in the development of sepsis.

## 1. Introduction

Sepsis is a life-threatening organ dysfunction caused by a dysregulated host response to infection.^[1]^ In patients with critical illness, it can lead to multiorgan dysfunction syndrome and circulatory collapse, which is a common clinical emergency.^[2]^ Approximately 31.5 million cases of sepsis and 19.4 million cases of severe sepsis are recorded in hospitals worldwide.^[3]^ According to a recently published article in China, sepsis affects one-fifth of patients admitted to the intensive care unit, and the 90-day mortality rate is approximately 35.5%.^[4]^

Sepsis is a complex series of immune responses to infection, and this condition is commonly associated with acquired immunodeficiency.^[5]^ Sepsis can lead to the activation of different cell types and the release of both proinflammatory and anti-inflammatory molecules.^[6]^ Therefore, early sepsis manifests mainly as a potent anti-infective response, whereas late sepsis manifests primarily as a violent and persistent immunosuppressive response. Neutrophils are the first line of defense against pathogens. In sepsis progression, the dysregulation of neutrophil is an important event.^[7]^ Thus, through the analysis of pathophysiology of how sepsis affects neutrophil dysfunction and delayed apoptosis, as well as the signaling pathways involved, may help us to develop new and promising therapeutic strategies for sepsis.

With the development and application of gene chip technology, the bioinformatics techniques have improved. Via secondary bioinformatics analysis of existing datasets, it is important to screen out genes that play a key role in different diseases, and further analysis and research should be performed.^[8]^ The current study aimed to identify genes that may play a key role in neutrophil dysfunction and delayed apoptosis during sepsis progression by comprehensively analyzing immune cell status based on data collected from Gene Expression Omnibus (GEO). The findings can be used as a basis for the construction of gene networks and the screening of potential key molecular targets, which may provide novel insights on the diagnosis, pathogenesis, and treatment of sepsis.

## 2. Materials and methods

### 2.1. Sepsis data collection

Sepsis datasets were downloaded from the GEO database (https://www.ncbi.nlm.nih.gov/geo/).^[9]^ The datasets of GSE28750^[10]^ and GSE64457^[11]^ were obtained. The GSE28750 dataset included whole blood samples collected from 20 healthy controls and 10 patients with sepsis. The GSE64457 dataset contained neutrophil samples collected from 8 healthy controls and 15 patients with sepsis. In this study, the GSE64457 dataset was used to screen DEGs and identify the hub genes in neutrophils in sepsis. The GSE28750 dataset was used to estimate the fraction of 24 types of immune cells in the whole blood in sepsis. The GSE28750 dataset was used to validate the hub genes. Data were downloaded from the GEO database using the R package “GEOquery.”^[12]^

### 2.2. Differential expression genes analysis

Gene expression profiles were constructed for each sample of sepsis patients and controls. Differential analysis between sepsis patients and healthy individuals in downloaded dataset was investigated using the R package “limma” version 3.42.2. The R package “ggplot2” version 3.3.3 was used to visualize the analysis results.^[13]^ An adjusted *P*-value of <.05 and |log2fold change| of ≥1 were used as the cutoff values.

### 2.3. Evaluation of immune cells in the whole blood

The current study used the Immune Cell Abundance Identifier (ImmuCellAI) website (http://bioinfo.life.hust.edu.cn/ImmuCellAI) to estimate the fraction of 24 types of immune cells in samples from the GSE28750 dataset. ImmuCellAI^[14]^ is an analytical tool used to estimate gene expression profiles and data to evaluate the abundance of member cell types in a mixed population of cells.

### 2.4. Function and pathway enrichments of differentially expressed mRNAs

Gene ontology (GO)^[15]^ analysis was performed on genes identified via differential analysis. The gene functions include cellular component, molecular function, and biological process (BP). These 3 major categories can explain the biological functions of genes at different levels, and genes can be annotated and classified via GO analysis to further validate the functions of the obtained differential genes and their enriched functions. Kyoto Encyclopedia of Genes and Genomes pathway analysis was used to identify enriched pathways and screen out the key signal pathways involved in a specific BP. The analysis and visualization of the differentially expressed genes (DEGs) were performed using the R package “ggplot2,” “clusterPorfiler,” and “enrichplot.”

### 2.5. Establishment of protein–protein interaction network and analysis of hub genes

To obtain and understand the interaction between differential genes, the obtained differential genes were analyzed using STRING (functional protein association networks http://string-db.org).^[16]^ The obtained differential genes were entered on the STRING website, and the minimum correlation score was set to 0.4. The obtained protein interaction network was further visualized using Cytoscape (3.6.1).^[17]^ The results were imported into Cytoscape, and the APP named MCODE was used to find the tightly connected regions in the interactive network diagram. The hub genes were identified according to the scores.

### 2.6. Cell culture and treatments

4ml of whole venous blood was taken from healthy people for physical examination. Human peripheral blood neutrophils were isolated and cultured by Ficoll density gradient centrifugation. A standard neutrophils cell medium (ECM) (ScienCell, San Diego, CA) was used to culture the cells and they were incubated at 37 °C under 5% CO_2_. Standard cell culture techniques were used to maintain the cells. The neutrophils were then treated with 1 μg/mL lipopolysaccharide (LPS) for 6 and 12 hours.

### 2.7. Quantitative real-time PCR

Following the manufacturer’s instructions, we isolated total RNA from cells using the TRIzol reagent (Invitrogen, Carlsbad, CA). Reverse transcriptase was added to the RNA sample according to the manufacturer’s instructions to reverse transcribe mRNA into cDNA.

### 2.8. Statistical analysis

Continuous variables with normal distribution were compared using the Student test or the Mann–Whitney *U* test. The correlations between continuous variables were assessed using the Spearman correlation coefficient. Given the nature of our biological data and the potential presence of nonlinear relationships and outliers, the Spearman correlation coefficient was chosen to ensure robust and reliable assessments of the relationships between continuous variables. Receiver operating characteristic curve (ROC) curves are used to evaluate the diagnostic accuracy of the identified genes, with the area under the curve serving as a measure of overall performance. ROC curves are threshold-independent, which means they provide a comprehensive evaluation of the diagnostic ability of the genes across all possible thresholds. Statistical analyses were performed using the Statistical Package for the Social Sciences 19.0 (IBM, Armonk, NY). A *P* value of <.05 was statistically significant.

## 3. Result

### 3.1. Identification of DEG and enrichment analysis of samples collected from patients with sepsis and normal controls in the GSE64457 dataset

**Figure 1.**
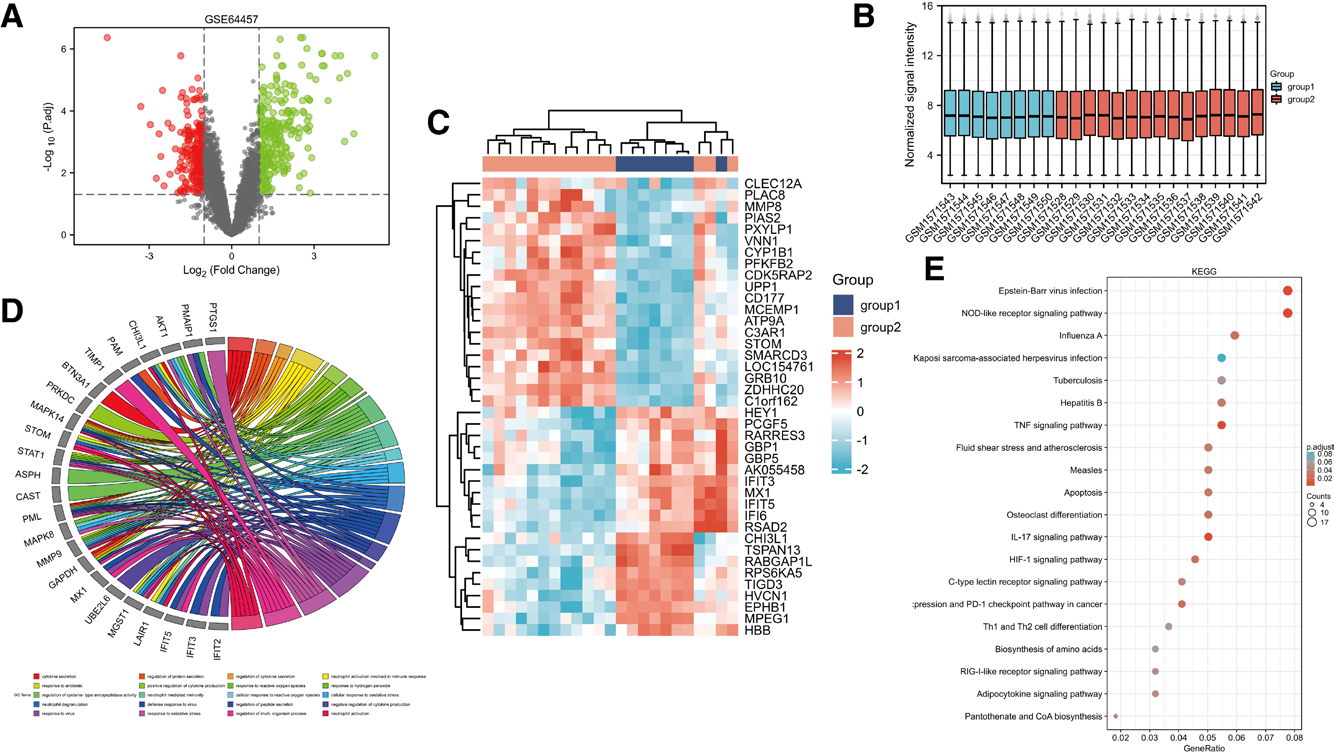
Differentially expressed gene and GO and KEGG pathway in the GSE64457 dataset. (A) Volcano map of the differentially expressed genes in the GSE64457 dataset. (B) Box plot of the GSE64457 dataset. (C) Heatmap of differentially expressed genes in the GSE64457 dataset. (D) Chord plot depicting the association between genes and Gene Ontology (GO) terms of biological process. (E) The top 20 KEGG pathway terms that were significantly enriched for the differentially expressed genes in the GSE64457 dataset. Group 1 included 8 samples from healthy controls, and group 2 comprised 15 samples from patients with sepsis. Differentially expressed mRNAs were screened using a LogFC threshold of ≥1.5 or ≤–1.5 and an adjusted *P*-value of <.05. The red dot represents the upregulated genes, and the green dots indicates the downregulated genes. KEGG = Kyoto Encyclopedia of Genes and Genomes.

DEGs in neutrophil samples collected from patients with sepsis and normal controls were analyzed using the “Limma” package. As shown in Figure 1A and C, 285 significantly upregulated genes and 204 significantly downregulated genes were identified. The gene microarray data of the sepsis and healthy control groups after logarithmic standard processing showed that the study specimens had better data homogeneity after logarithmic processing, and the results were comparable between the 2 groups (Fig. 1B). Based on the GO analysis of 489 DEGs, genes were mainly involved in BPs associated with neutrophil activation and neutrophil degranulation (Fig. 1D and Table 1). Kyoto Encyclopedia of Genes and Genomes analysis of 489 DEGs showed that genes were mainly involved in the pathway associated with the TNF signaling and apoptosis pathway (Fig. 1E).

**Table 1:** Gene ontology (GO) terms and KEGG pathway terms.

Ontology	ID	Description	GeneRatio	BgRatio	pvalue	p.adjust	qvalue
BP	GO:0042119	Neutrophil activation	51/410	498/18,670	3.28e‐20	1.42e‐16	1.17e‐16
BP	GO:0043312	Neutrophil degranulation	49/410	485/18,670	3.38e‐19	6.33e‐16	5.19e‐16
BP	GO:0002283	Neutrophil activation involved in immune response	49/410	488/18,670	4.39e‐19	6.33e‐16	5.19e‐16
BP	GO:0002446	Neutrophil mediated immunity	49/410	499/18,670	1.12e‐18	1.21e‐15	9.91e‐16
BP	GO:0009615	Response to virus	32/410	326/18,670	1.50e‐12	1.30e‐09	1.07e‐09
CC	GO:0042581	Specific granule	28/419	160/19,717	6.36e‐18	2.85e‐15	2.50e‐15
CC	GO:0035580	Specific granule lumen	17/419	62/19,717	8.21e‐15	1.84e‐12	1.61e‐12
CC	GO:0070820	Tertiary granule	24/419	164/19,717	9.62e-14	1.44e-11	1.26e-11
CC	GO:1904724	Tertiary granule lumen	14/419	55/19,717	6.08e‐12	6.83e‐10	5.97e‐10
CC	GO:0060205	Cytoplasmic vesicle lumen	31/419	338/19,717	8.88e‐12	7.17e‐10	6.27e‐10
KEGG	hsa04621	NOD-like receptor signaling pathway	17/219	181/8076	7.55e‐06	0.002	0.002
KEGG	hsa05169	Epstein-Barr virus infection	17/219	202/8076	3.20e‐05	0.003	0.003
KEGG	hsa04657	IL-17 signaling pathway	11/219	94/8076	4.34e‐05	0.003	0.003
KEGG	hsa04668	TNF signaling pathway	12/219	112/8076	4.74e‐05	0.003	0.003
KEGG	hsa05235	PD-L1 expression and PD-1 checkpoint pathway in cancer	9/219	89/8076	6.51e‐04	0.025	0.022

BP = biological process, CC = cellular component, KEGG = Kyoto Encyclopedia of Genes and Genomes.

### 3.2. Identification of hub-genes using the PPI network

**Figure 2.**
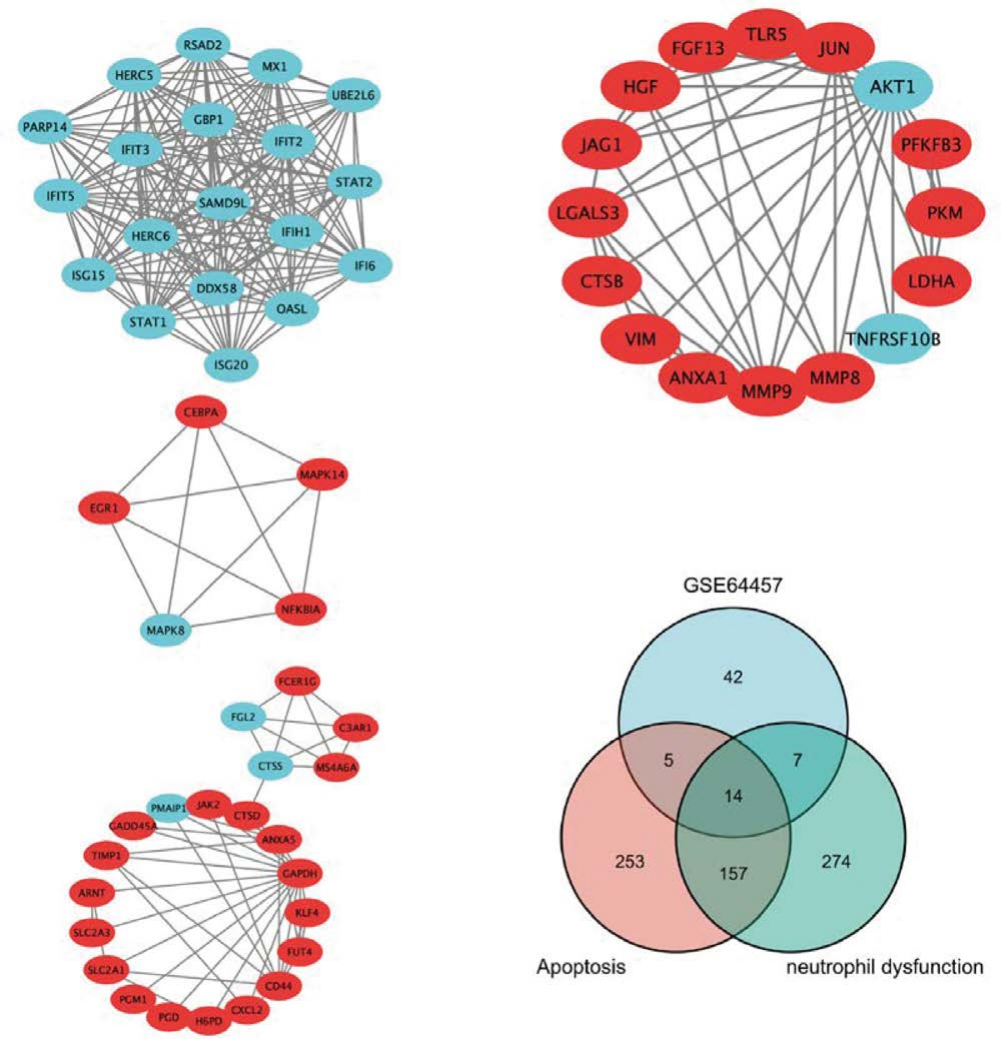
Protein–protein interaction networks of DEGs in the GSE64457 dataset. The most significant module was obtained from the PPI network using Cytoscape. The Venn diagram showing overlapping genes between the hub genes of GSE64457 and apoptosis- and neutrophil dysfunction-related genes obtained from GeneCards. The blue color indicates the downregulated hub genes, and the red represents the upregulated hub genes. In total, 14 hub genes were identified in the overlapping region.

The 489 DEGs were subjected to PPI analysis using STRING, and the obtained protein interactions network was further analyzed using Cytoscape to find tightly connected regions in the interactions network map and find key genes based on the score by applying MCODE. Based on the GeneCards (https://www.genecards.org), data on genes associated with apoptosis and neutrophil activation were obtained. Genes with a score of >20. The intersection was taken between DEGs and apoptosis- and neutrophil dysfunction-related genes using Venn diagrams (Fig. 2).

### 3.3. Identification of DEGs collected from patients with sepsis and normal controls in the GSE28750 dataset

**Figure 3.**
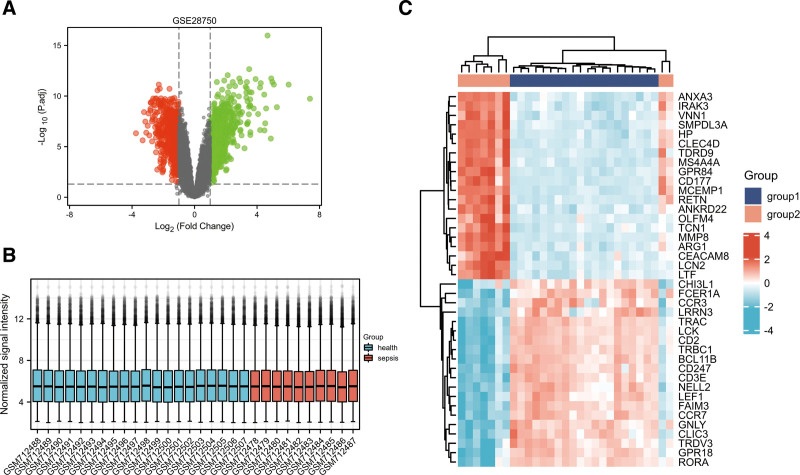
Differentially expressed genes in the GSE28750 dataset. (A) Volcano map of the differentially expressed genes in the GSE28750 dataset. (B) Box plot of the GSE28750 dataset. (C) Heatmap of differentially expressed genes in the GSE28750 dataset. Group 1 included 20 samples collected from normal healthy controls, and group 2 comprised 9 samples collected from patients with sepsis. Differentially expressed mRNAs were screened using a LogFC threshold of ≥1.5 or ≤–1.5 and an adjusted *P*-value of <.05. The red dot indicates the upregulated genes, and the green dots represents the downregulated genes.

DEGs in the whole blood samples collected from patients with sepsis and normal controls were analyzed using the “Limma” package. As shown in Figure 3A and C, 346 significantly upregulated genes and 225 significantly downregulated genes were identified. The gene microarray data of the sepsis and healthy control groups after logarithmic standard processing showed that the study specimens had better data homogeneity after logarithmic processing and were comparable between the 2 groups (Fig. 3B).

### 3.4. Immune cell infiltration associated with the characteristics of patients with sepsis

**Figure 4.**
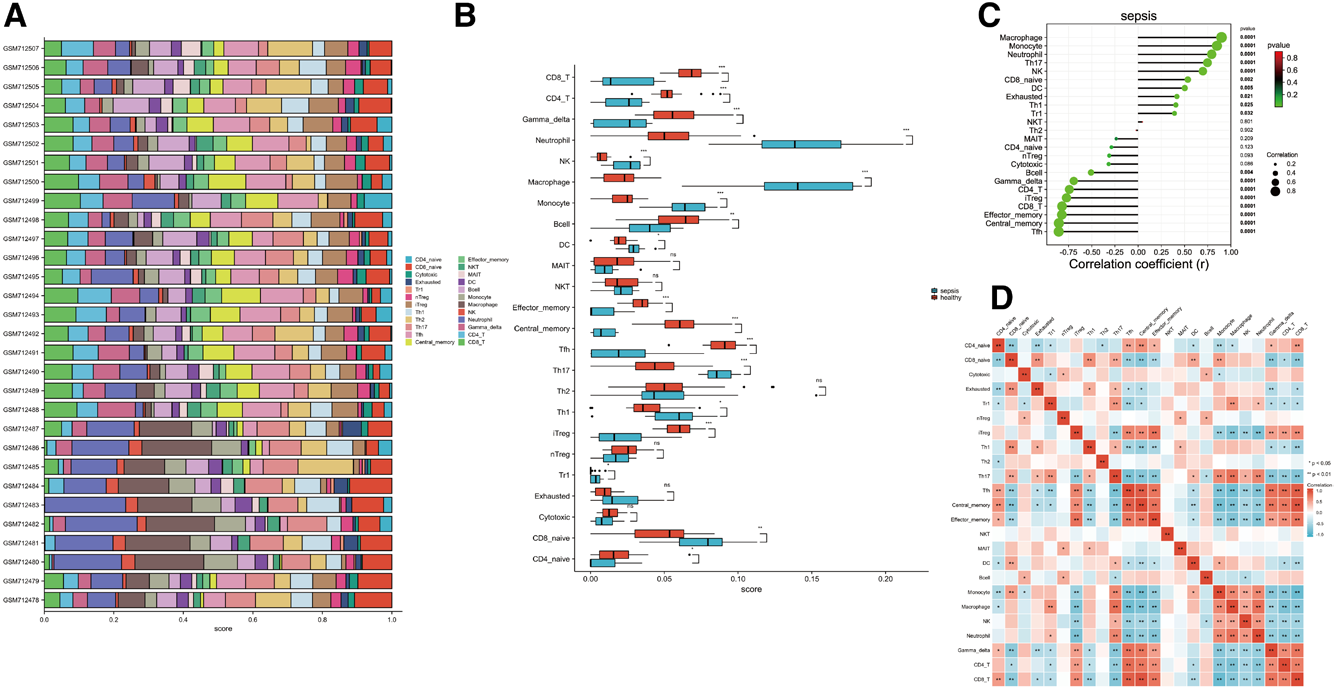
Immune cell distribution in patients with sepsis. (A) Differences in immune cell infiltration between patients with sepsis and normal healthy controls. Each row represents a sample from the GSE28750 dataset. Each color indicates a type of immune cell. (B) Box plot shows the distribution of 24 types of immune cells in normal controls and patients with sepsis in the GSE28750 dataset. (C) The association between sepsis and immune cell infiltration level. (D) Correlation between different immune infiltrating cells in sepsis based on Pearson correlation analysis. The red and blue nodes represent positive and negative correlations. *P < .05, **P < .01.

The disorder of immune function plays an important role in sepsis progression. Therefore, we assessed immune cell infiltration in patients with sepsis. To explore immune cell infiltration in patients with sepsis and healthy controls, the DEGs from the GSE28750 dataset were analyzed. ImmuCellAI was used to estimate the fraction of 24 types of immune cells in the samples from the GSE28750 dataset. Figure 4A and B show the distribution of 24 types of immune cells in the samples from the GSE28750 dataset. Results showed that macrophages, monocytes, neutrophils, natural killer (NK) cells, T helper 17 cells, and dendritic cells were upregulated, and CD8+ T cells, CD4+ T cells, T-regulatory cells, and T follicular helper cells (TFH) were downregulated in the whole blood of patients with sepsis.

Next, the association between immune infiltration and sepsis was analyzed. As shown in Figure 4C, the infiltration level of neutrophils (*R* = 0.793, *P* < .0001), monocytes (*R* = 0.849, *P* < .0001), macrophages (*R* = 0.899 *P* < .001), Th17 (*R* = 0.747, *P* < .0001), and NK (*R* = 0.698, *P* < .0001) was positively correlated with sepsis. The infiltration level of CD4+ T cells (r = −0.741, *P* < .0001), CD8+ T cells (r = −0.82, *P* < .0001), and TFH (r = −0.856, *P* < .0001) were negatively correlated with sepsis. Based on the correlation heatmap (Fig. 4D), neutrophils were significantly positively correlated with macrophages, monocytes, and NK cells. In contrast, neutrophils had a significant negative correlation with CD4 and CD8 cells.

### 3.5. Selection and validation of hub genes

**Figure 5.**
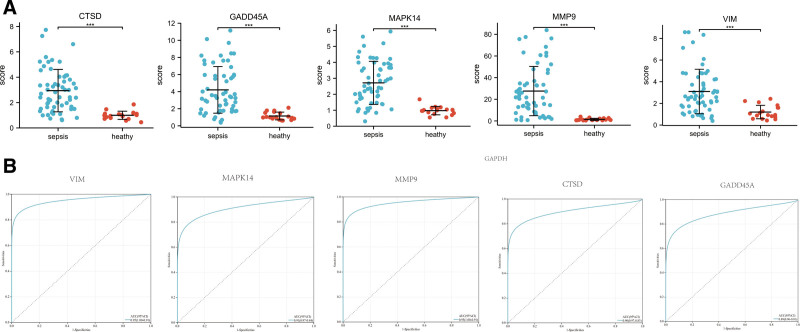
(A) Selection and validation of the top 6 hub genes. The expression levels of CTSD (*P* < .001), GADD45A (*P* < .001), MAPK14 (*P* < .001), MMP9 (*P* < .001), and VIM (*P* < .001) were significantly higher in patients with sepsis than in normal health controls. (B) Receiver operating characteristic curve analysis of top 6 hub genes. The area under the curves of CTSD, GADD45A, MAPK14, MMP9, and VIM were 0.90 (0.83–0.97), 0.89 (0.81–0.96), 0.91 (0.84–0.87), 0.95 (0.91–1.00), 0.90 (0.83–0.97), and 0.95 (0.91–1.00).

Five of 14 hub genes were selected according to the adjusted *P* value. These genes included CTSD, GADD45A, MAPK14, MMP9, and VIM. The expression levels of these genes in the GSE28750 dataset were first validated. As shown in Figure 5A, the expression levels of CTSD (*P* < .001), GADD45A (*P* < .001), MAPK14 (*P* < .001), MMP9 (*P* < .001), and VIM (*P* < .001) were significantly higher in patients with sepsis than in normal healthy controls. As shown in Table 2, the expression levels of these 6 hub genes did not significantly differ in GSE83824^[18]^ (macrophages) and GSE46955^[19]^ (monocytes) between sepsis and health controls.

**Table 2:** The differential expression of hub-genes in GSE83824 and GSE46955.

Gene symbol	LogFC	p.adjust	Dataset
CTSD	‐0.737703994	0.304801807	GSE83824
GADD45A	0.251749456	0.05983702	GSE83824
MAPK14	‐0.373704152	0.005293752	GSE83824
MMP9	‐0.145299897	0.324862324	GSE83824
GAPDH	0.114340517	0.30498879	GSE83824
VIM	0.592944677	0.000843272	GSE83824
CTSD	‐0.280049513	0.477684948	GSE46955
GADD45A	0.297127884	0.770760869	GSE46955
MAPK14	‐0.436699416	0.276345634	GSE46955
MMP9	‐0.173223593	0.883507913	GSE46955
GAPDH	‐0.533537935	0.424505972	GSE46955
VIM	‐0.007354785	0.95982961	GSE46955

The ability of the abovementioned genes for predicting the prognosis of sepsis was evaluated using the ROC analysis. As shown in Figure 5B, the area under the curves of CTSD, GADD45A, MAPK14, MMP9, and VIM were 0.90 (0.83–0.97), 0.89 (0.81–0.96), 0.91 (0.84–0.87), 0.95 (0.91–1.00), 0.90 (0.83–0.97), and 0.95 (0.91–1.00), respectively.

**Figure 6.**
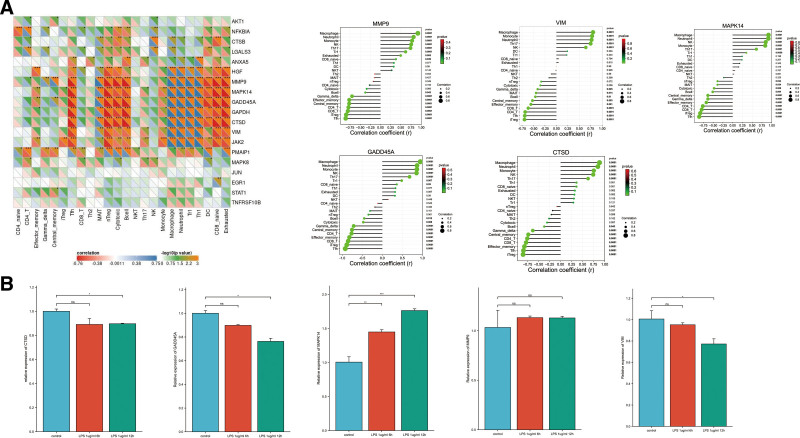
(A) Correlation between the key genes and immune infiltrating cells based on Pearson correlation analysis. The red and blue nodes represent positive and negative correlations. *P < .05, **P < .01. (B) Expression of VIM, CTSD, MMP9, MAPK14, and GADD45A in neutrophils treated with LPS. LPS = lipopolysaccharide.

The association between hub gene expression and immune infiltration in patients with sepsis patients was analyzed. As shown in Figure 6A, the infiltration levels of neutrophils, monocytes, macrophages, Th17, and NK were positively correlated with the expression of the 6 hub genes. The infiltration levels of CD4+ T cells, CD8+ T cells, and TFH were negatively correlated with the expression of the 6 hub genes.

These 5 DEGs with higher degree including CTSD, GADD45A, MAPK14, MMP9, and VIM were selected as candidate according to the bioinformatics analyses. The expression level of MAPK14 was significantly upregulated in the neutrophils induced by LPS compared with controls (Fig. 6B). The expression level of CTSD, GADD45A, MMP9, and VIM in neutrophils induced by LPS was not consistent with our bioinformatics analysis.

## 4. Discussion

Sepsis is a common critical illness with a high mortality rate. Further, it is a complex pathological process in which pathogenic microorganisms act as the initiating factor, followed by the release of pro- and anti-inflammatory cytokines from a variety of immune cells.^[20]^ Neutrophils are the first line of defense, and they protect the body against the progression of pathogenic infections. Failure of neutrophils at the site of infection and dysregulation of the immune response at the onset of sepsis are the important mechanisms of sepsis progression.^[21]^ Therefore, it is important to identify potential molecules involved in neutrophil dysfunction could help identify early sepsis and drugs that prevent the development of sepsis.

The current study aimed to identify the potentially relevant molecules associated with neutrophil dysfunction during sepsis progression via bioinformatic analysis of sepsis-related datasets. In the initial analysis, DEGs and PPI were combined to identify potential hub genes from neutrophil samples in the GSE64457 dataset. Five hub genes including CTSD, GADD45A, MAPK14, MMP9, and VIM were selected for further analysis based on the independent GSE28750 dataset with peripheral blood samples. The expression of these hub genes was not significant in macrophage (GSE83824) and monocyte (GSE46955) samples. Mechanistically, these hub genes may function via several immune-related pathways and neutrophils. In addition, according to the ROC curve, the expression of these hub genes may facilitate the diagnosis of sepsis. The hub genes may influence the dysfunction of neutrophils, which can induce the development of sepsis. Several studies have identified the biomarkers of sepsis based on gene expression profiles from public databases. Zeng et al,^[22]^ found that MAPK14, FGR, RHOG, LAT, PRKACB, UBE2Q2, ITK, IL2RB, and CD247 were associated with immune dysregulation and sepsis development.

Our study analyzed differences in immune infiltrating cells between sepsis peripheral blood samples and normal peripheral blood samples. A previous study evaluated immune cell infiltration in patients with sepsis. Results showed that the infiltration levels of macrophages, monocytes, and neutrophils were significantly higher, which was significantly correlated with sepsis. Under normal circumstances, neutrophils are essential for the early control of invasive pathogens.^[23]^ In patients with sepsis, the higher expression of circulating neutrophils of different maturity levels is caused by the increased release of immature neutrophils and delayed apoptosis of circulating neutrophils.^[24]^ If sepsis develops, their migration and antimicrobial activity are impaired, which contribute to the dysregulation of immune response. Meanwhile, neutrophils produce large amounts of interleukin-10 during sepsis development.^[25]^ In an experiment using a human model of endotoxemia in which healthy volunteers received low-dose endotoxin, neutrophils suppressed T cell proliferation.^[26]^ These cells may influence the function of macrophages and monocytes during sepsis progression. Die to response to infection, neutrophils are recruited in the infectious sites to reduce macrophage pyroptosis, which then influences sepsis progression after bacterial infection.^[27]^ Yang revealed that exosomal miR-30d-5p from neutrophils contributed to sepsis-related acute lung injury by inducing macrophage polarization and pyroptosis.^[28]^ Based on this study, neutrophils were significantly positively correlated with macrophages, monocytes, and NK cells. In contrast, neutrophils were significantly negatively correlated with CD4 and CD8 cells. In sepsis progression, the function of almost all types of immune cells is directly and indirectly impaired.

Five hub genes including CTSD, GADD45A, MAPK14, MMP9, and VIM were upregulated in patients with sepsis compared with normal controls in the GSE28750 (peripheral blood samples) and GSE64457 (neutrophil samples) datasets. The expression levels of these genes did not significantly differ in GSE83824 (macrophages samples) and GSE46955 (monocytes samples) between patients with sepsis and health controls. Meanwhile, these genes were positively correlated with the infiltration levels of neutrophils, monocytes, macrophages, and Th17 and NK cells. The infiltration levels of CD4+ T cells, CD8+ T cells, and TFH were negatively correlated with the expression of the 6 hub genes. The expression of these genes in neutrophils may play an important role in neutrophil dysfunction and immune disorders during the development of sepsis. Further, these genes can predict the prognosis of sepsis according to ROC analysis. In vivo and in vitro molecular experiments should be performed in the future, and follow-ups with larger clinical samples should be conducted to validate our main results. According to the result of RT-PCR, only MAPK14 was significantly upregulated compared with controls, which was concordant with the bioinformatics analysis results. MAPK14 has been shown to play a crucial role in the inflammatory response and immune regulation. Its significant upregulation in sepsis patients, as validated by RT-PCR, suggests that it could serve as a reliable biomarker for early diagnosis. Early detection of sepsis is critical for timely intervention and improved patient outcomes. By measuring the expression levels of MAPK14 in peripheral blood samples, clinicians can potentially identify patients at risk of developing sepsis or those in the early stages of the disease. This could enable prompt initiation of appropriate treatments, thereby reducing morbidity and mortality rates. The dysregulation of MAPK14 in neutrophils during sepsis progression highlights its potential as a therapeutic target. Neutrophils are key players in the innate immune response, and their dysfunction contributes significantly to the pathogenesis of sepsis. Targeting MAPK14 could help restore neutrophil function and mitigate the excessive inflammatory response observed in sepsis. Therefore, further study of MAPK14 about function in the pathophysiology of sepsis is needed to confirm our hypothesis.

The current study had several limitations. First, the current analysis is limited by the size and diversity of the datasets. Larger and more diverse patient cohorts should be included in future studies to better generalize the findings across different populations and clinical settings. This will also help in validating the diagnostic and therapeutic value of the identified genes in various contexts. Second, although the study has conducted preliminary experimental validation through RT-PCR for MAPK14, further in-depth functional studies are necessary. In vitro experiments using cell lines and primary neutrophils, as well as in vivo studies using animal models, can provide direct evidence of the roles of the identified genes in neutrophil dysfunction and immune disorders. Techniques such as CRISPR/Cas9 for gene editing, RNA interference, and overexpression can be employed to manipulate gene expression and observe the effects on cellular behavior and disease progression. Thirdly, understanding the precise mechanisms by which these genes influence neutrophil function and immune responses is crucial. Future research should focus on elucidating the signaling pathways and molecular interactions involved. For example, studying the downstream targets of these genes and their interactions with other signaling molecules can provide deeper insights into their roles in sepsis.

## 5. Conclusion

This study has identified 5 key genes – CTSD, GADD45A, MAPK14, MMP9, and VIM – that are significantly upregulated in sepsis patients compared to healthy controls. Through comprehensive bioinformatics analysis, including differential expression analysis, protein–protein interaction analysis, and immune infiltration assessment, we have provided strong evidence that these genes play crucial roles in neutrophil dysfunction and immune disorders during sepsis progression. The high diagnostic accuracy of these genes, as demonstrated by the area under the ROC curve, suggests their potential as valuable biomarkers for early detection and prognosis of sepsis.

The identification of MAPK14, in particular, highlights its significance as a therapeutic target. Its upregulation in sepsis patients and validation through RT-PCR underscore its role in the dysregulation of neutrophil function and the excessive inflammatory response. Targeting MAPK14 could offer new therapeutic strategies to restore immune balance and improve patient outcomes. Potential approaches include pharmacological inhibition, gene therapy, and biological therapies, all of which warrant further investigation in preclinical and clinical settings.

Moreover, the findings of this study contribute to the broader understanding of sepsis pathogenesis by elucidating the molecular mechanisms underlying neutrophil dysfunction. By integrating these insights with clinical data, we can develop more personalized and effective treatment plans. The use of these biomarkers in clinical practice could facilitate early intervention and tailored therapies, ultimately leading to better management and reduced mortality rates in sepsis patients.

In summary, this study not only advances our knowledge of the genetic and molecular underpinnings of sepsis but also provides a solid foundation for future translational research. Further validation in larger and more diverse patient cohorts is necessary to fully realize the clinical potential of these findings. We anticipate that our work will inspire ongoing efforts to improve the diagnosis, treatment, and prevention of sepsis, thereby enhancing patient care and outcomes.

## Author contributions

**Data curation:** Junfeng Zhang.

**Formal analysis:** Junfeng Zhang.

**Investigation:** Qinghui Fu.

**Methodology:** Junfeng Zhang, Qinghui Fu, Jianfeng Zhao.

**Project administration:** Qinghui Fu, Jianfeng Zhao.

**Resources:** Jianfeng Zhao.

**Software:** Jianfeng Zhao.

**Supervision:** Qinghui Fu.

**Validation:** Junfeng Zhang.

**Visualization:** Junfeng Zhang, Qinghui Fu.

**Writing – original draft:** Junfeng Zhang.
